# Urgency for standardized protocols to improve clinical implementation of artificial intelligence in endoscopic diagnostics

**DOI:** 10.1055/a-2695-2841

**Published:** 2025-09-24

**Authors:** Ulrik Deding, Benedicte Schelde-Olesen, Ervin Toth, Anastasios Koulaouzidis, on behalf of the AICE consortium

**Affiliations:** 1 6174 Department of Clinical Research, University of Southern Denmark, Odense, Denmark; 2 11286 Department of Surgery, Odense University Hospital, Svendborg, Denmark; 3 11286 Surgical Research Unit, Odense University Hospital, Odense, Denmark; 4 Endoscopy Unit, Department of Gastroenterology, Skane University Hospital, Malmo, Sweden; 5 37805 Department of Social Medicine & Public Health, Pomeranian Medical University in Szczecin, Szczecin, Poland

The recent systematic review by Cold et al. 1 offers a comprehensive review of artificial intelligence (AI) tools designed for preparation scoring in colonoscopy. Despite confirming high performance, the review highlights significant limitations such as inconsistent validation methods, a lack of external reproducibility, minimal integration into clinical workflows, and suboptimal reference standards. Most importantly, correlation with clinically meaningful endpoints such as adenoma detection rate (ADR) or adenoma miss rate (AMR) is scarce, raising concerns about practical impact on patient outcomes. This is particularly problematic when the benchmark itself (colonoscopy) has a mean AMR of 26%, raising serious concerns about its suitability as a reference standard for validating AI systems, especially when those systems are applied to other modalities such as capsule endoscopy (CE), where the diagnostic context is fundamentally different.

Although interobserver agreement using the Boston Bowel Preparation Score (BBPS) may be high, it holds little clinical value if not linked to hard outcomes. Moreover, training AI on only high-consensus images introduces bias, making models less effective in ambiguous or difficult cases. Although it streamlines annotation, it biases AI toward clear-cut cases, reducing performance in diagnostically challenging images. Clinical-grade AI must be trained on both ambiguous and consensus cases to ensure balanced diagnostic capability.

Nadimi et al. 2 address these gaps with an explainable AI (XAI) model integrated into the colon CE (CCE) workflow, automating polyp detection, characterization, and sizing. Using methods such as GradCAM++, CartoonX, and Pixel RDE, the system combines technical strength with transparency to support clinical trust and adoption. Their work highlights the value of combining AI tools to achieve more precise and detailed results, aligning with the discussion by Cold et al. In parallel, Mascarenhas-Saraiva et al. 3 developed a rule-based AI for bowel prep assessment in CCE, prioritizing interpretability and real-time feedback. Designed for clinical use, it aims to enhance trust and reduce fatigue or variation in high-volume endoscopy settings.

Most current AI models in capsule endoscopy ignore key clinical cues such as frame and pixel clustering and time spent on specific regions. This lack of temporal modeling limits diagnostic realism and disconnects AI behavior from clinician reasoning. Although less critical in live colonoscopy, it is essential in asynchronous CCE. In addition, Moen et al. 4 outlined critical challenges and future directions for AI in CCE. Their review emphasizes the need for harmonized datasets, transparent AI models, and coordinated clinical validation. These systemic challenges support the rationale for a unified framework such as The Artificial Intelligence in Capsule Endoscopy (AICE) project to ensure alignment between technological development and clinical implementation.

AICE was funded by EU to address precisely these issues. AICE is a European collaboration committed to building a cohesive and clinically driven framework for AI implementation in CCE. It aims to standardize evaluation protocols, support multicenter validation efforts, and advocate for alignment with established international standards, including those of the European Society of Gastrointestinal Endoscopy and the World Endoscopy Organization. By fostering collaboration between clinicians, data scientists, and regulatory stakeholders, AICE seeks to transform AI from a set of isolated tools into an integrated component of endoscopic care. AICE also prioritizes explainability and usability as core pillars of responsible AI. The initiative supports use of interpretable models, transparent reporting guidelines, and validation in real-world settings. This approach ensures that end-users, especially clinicians, can trust and effectively interact with AI outputs. It also lays the foundation for regulatory approval and integration into national screening programs, particularly as health systems become more digitally mature.

However, despite notable individual advances, the broader field of AI in endoscopy remains fragmented. There is still limited consensus on validation protocols, outcome measures, reference standards, and implementation standards 5 . It looks as if the momentum of innovation has outpaced the frameworks for adoption. What is needed now is alignment on reporting, external validation across populations, and endpoints that reflect real patient outcomes, including diagnostic uncertainty, not just retrospective accuracy.

**Publication note**

Letters to the editor do not necessarily represent the opinion of the editor or publisher. The editor and publisher reserve the right to not publish letters to the editor, or to publish them abbreviated or in extracts.
